# How do users of a mental health app conceptualise digital therapeutic alliance? A qualitative study using the framework approach

**DOI:** 10.1186/s12889-025-23603-5

**Published:** 2025-07-14

**Authors:** Theresa Taylor, Simon D’Alfonso, Maria João Tralhão Dolan, Jenny Yiend, Pamela Jacobsen

**Affiliations:** 1 Department of Psychology, University of Bath, Bath, UK; 2 School of Computing and Information Systems, University of Melbourne, Melbourne, Australia; 3 Institute of Psychiatry, Psychology and Neuroscience, King’s College London, London, UK

**Keywords:** Therapeutic alliance, Digital therapeutic alliance, Persuasive systems design, Self-guided mental health smartphone application, Unguided digital intervention, Mobile phone

## Abstract

**Background:**

Self-guided mental health smartphone applications (apps) have the potential to increase access to evidence-based psychological interventions and reduce the burden on staff resources in strained mental health services. Within human-delivered therapy, the working relationship (therapeutic alliance) between the client and therapist is well studied and has been consistently linked to effective and engaging therapy. However, less is known about whether a digital therapeutic alliance exists, what its components may be, and how it can be fostered to improve engagement and adherence to smartphone applications. This study explored the experiences of users of a mental health app to better understand digital therapeutic alliance and how persuasive systems design may help us understand which features of app design influence this relationship.

**Methods:**

We conducted a qualitative study using semi-structured interviews with 13 participants who had recent experiences of using the STOP app which targets paranoia. Data were analyzed using framework analysis with therapeutic alliance and persuasive systems design as deductive theoretical frameworks.

**Results:**

We constructed five dimensions of digital therapeutic alliance: 1) Humanness of the app, 2) Personal meaningfulness, 3) Progression towards goals, 4) How is it to use the app, and 5) Flexibility enhances relationship. Themes 1–4 map onto the existing dimensions of therapeutic alliance, and Theme 5 (Flexibility enhances relationship) provides the context within which a digital therapeutic alliance forms. Persuasive systems design features were found to reinforce and enhance aspects of digital therapeutic alliance.

**Conclusions:**

This study provides valuable insight into the existence of digital therapeutic relationships (alliance) and its dimensions. From our findings, there are indicators that digital therapeutic alliance is a digital analogue of therapeutic alliance and is enhanced by persuasive features of the app. Findings from this study could be used to inform the design of mental health apps to enhance their capacity to foster digital therapeutic alliance with users, with the supposition that as with the traditional therapeutic alliance, its digital counterpart is also conducive to better efficacy in mental health apps.

**Supplementary Information:**

The online version contains supplementary material available at 10.1186/s12889-025-23603-5.

## Background

There is a well-recognised global gap between the need for and provision of services to prevent, identify and treat mental health conditions. In response, digital solutions have become a critical target for increasing availability of psychological care over the last two decades [1]. Self-guided mental health smartphone applications (mental health apps), where no therapist input is required, have the potential to reduce demand on under-resourced mental health services [2]. Yet, efficacy data for treating mental health symptoms using apps without clinician support is still emerging. A recent meta-analysis of 176 RCTs found that self-guided apps have overall small but significant effects on symptoms of depression and generalized anxiety. Nevertheless, results for other conditions were less clear, with a small effect being found on post-traumatic stress symptoms and a non-significant negative effect found on panic symptoms [3]. Additionally, adherence to mental health apps is low in both trial and real-world settings. For example, a systematic review of 93 publicly available mental health and well-being apps found that only 3.3% (IQR 6.2%) of users were still accessing the app after 30 days [4]. Similarly, a systematic review and meta-analysis of 70 RCTs of mental health apps found that adherence declined with time [5]. One reason for poor engagement and limited efficacy of self-guided mental health apps may be an insufficient therapeutic alliance [6].

### Therapeutic alliance

In human-delivered interventions**,** the quality of the relationship between the client and therapist, known as therapeutic alliance, is well studied and consistently linked to effective and engaging therapy [7, 8]. The most widely used theory is Bordin’s [9] pan-theoretical model of alliance, which defines alliance as consisting of goals (mutual understanding of what the client hopes to achieve), tasks (steps the therapist and client agree need to be taken towards the goal), and bond (trust and confidence between the client and therapist). Bordin [9] (pg. 252) stated that the therapeutic alliance was between a person seeking change and a “change agent” and could happen outside of therapy, suggesting potential applicability to smartphone applications. For example, a recent study used the Working Alliance Inventory (WAI) measure of therapeutic alliance to examine alliance with a self-guided app and found evidence of increasing alliance as the intervention progressed, comparable to ratings from studies in human-delivered psychotherapy [10].

### Digital therapeutic alliance

Building on traditional therapeutic alliance concepts, researchers have developed measures specifically for digital contexts. These measures include the Mobile Agnew Relationship Measure (mARM) [11] and the Digital Working Alliance Inventory (DWAI) [12], both based on existing therapeutic alliance measures (the Agnew Relationship Measure [ARM] and the WAI). Nevertheless, researchers have critiqued these measures, arguing that they do not sufficiently reconceptualize therapeutic alliance for the digital context, as they do not address the unique aspects of human-technology relationships [13]. Importantly, digital therapeutic alliance is conceptually and operationally distinct from digital product satisfaction, which is measured using the user experience questionnaire (UEQ) [14] and quantifies users’ experience of the application, such as their perception of its attractiveness, ease of use, and creativity.

Despite these measurement developments, the links between alliance and clinical outcomes in the context of self-guided mental health apps remain unclear [15, 16]. These mixed findings stand in contrast to human-delivered therapy, where more consistent associations between therapeutic alliance and clinical outcomes have been found. This lack of replication may be due to conceptual ambiguity or imprecise measurement of digital therapeutic alliance.

The uncertainty may stem from fundamental differences in how alliance operates in digital versus human contexts. It is possible that not all aspects of the traditional therapeutic alliance apply in the digital environment, or that digital therapeutic alliance may have unique dimensions salient to its context and features. For example, bond is considered crucial in therapeutic alliance; however, whether a person can build a bond with an app [13, 16, 17] is unclear. The importance of the alliance dimension of goals is also debated [13, 18], while there is some evidence that tasks are predictive of digital therapeutic alliance [16, 18, 19]. Conversely, research has pointed to unique dimensions of digital therapeutic alliance including flexibility [20], and users experiencing the app as less judgemental and pressurising than a human therapist [13, 16].

### Persuasive systems design

Given these conceptual challenges, there is a need to better understand, define and measure alliance in digital contexts by examining the unique attributes of technology itself. One promising approach draws from Human–Computer Interaction (HCI), a multidisciplinary field which explores the interface between digital technologies and people and designs technologies that allow humans to interact with machines in novel ways. HCI theories can therefore be useful to mental health professionals in understanding the possibility of a bi-directional relationship between an app and its user.

A particularly relevant subset of HCI for app developers is persuasive systems design, which involves using digital technologies to influence people’s behaviours and attitudes without using deception or coercion. A framework has been developed which transforms persuasive principles into software requirements and system features [21]. For example, an app feature such as praising users after they complete a session is a system feature of the persuasive systems design category “Dialogue Support”, which pertains to the feedback an interactive system provides users to help them move toward their goal. Authors have hypothesised that persuasive features may be one of the mechanisms through which digital therapeutic alliance develops [20, 22]; however, this has not been tested.

Understanding digital therapeutic alliance could therefore be essential to pinpointing mechanisms of effectiveness and engagement. The degree of overlap between therapeutic alliance and digital therapeutic alliance, and digital therapeutic alliance’s unique features, remains unclear. Moreover, there is a gap in the literature for studies that conceptualise alliance in the context of app features and design. This study will investigate whether and to what extent users perceive an alliance with a self-guided mental health smartphone application. It will also explore how aspects of persuasive systems design map onto user experience of an alliance with the application.

## Research questions

1. How do mental health smartphone application users conceptualise digital therapeutic alliance?
2. How does user experience of digital therapeutic alliance map onto persuasive systems design theory?

## Methods

This qualitative study used a phenomenological study design, approached from a critical realist epistemological position. The analysis was conducted using Framework Analysis. This report was written with reference to the 32-item Consolidated Criteria for Reporting [23] and Standards for Reporting Qualitative Research (SRQR) [24]. Recruitment was via approaching people who had completed their participation in a clinical trial of a self-guided smartphone application for mental health (Successful Treatment of Paranoia (STOP) trial) [25].

### The STOP trial

STOP trial participants were randomized into 3 arms: a 6-week intervention arm, a 12-week intervention arm, and a control arm, in addition to usual treatment [25]. The control arm participants received the same smartphone application as the intervention arms but with non-active content. Control participants performed the same reading and word-completion tasks with similar stories but without the therapeutic content designed to change paranoid thinking patterns. Both completers and non-completers of the STOP trial were invited to a the 24-week follow-up. STOP trial recruitment was from a range of local sources including NHS settings, research registers, service users’ networks, and voluntary sector organisations. Participants were eligible for the STOP trial if they were (i) aged 18 to 65 years; (ii) had a diagnosis featuring clinically significant persecutory or paranoid symptoms, present for a minimum of a month; (iii) scored 3 or more on the paranoia item (item 6) of the Positive and Negative Symptoms Scale (PANSS); (iv) scored 1 or less on the interpretation bias item (item 8) on the Similarity Ratings Task (SRT); (v) were stable on any psychotropic medication for a minimum of 3 months prior and were expected to remain so for the study duration; and (vi) were judged to have capacity to consent as assessed by a clinician. Participants were ineligible if they (i) had severe cognitive impairment; (ii) had illiteracy; (iii) had major physical illness (cancer, heart disease, stroke); (iv) had major substance or alcohol misuse as assessed by the SCID-V screen 5; or (v) were currently receiving, due to receive, or had received in the last 3 months a psychological intervention targeting paranoid beliefs.

### Intervention description

**Fig. 1:**
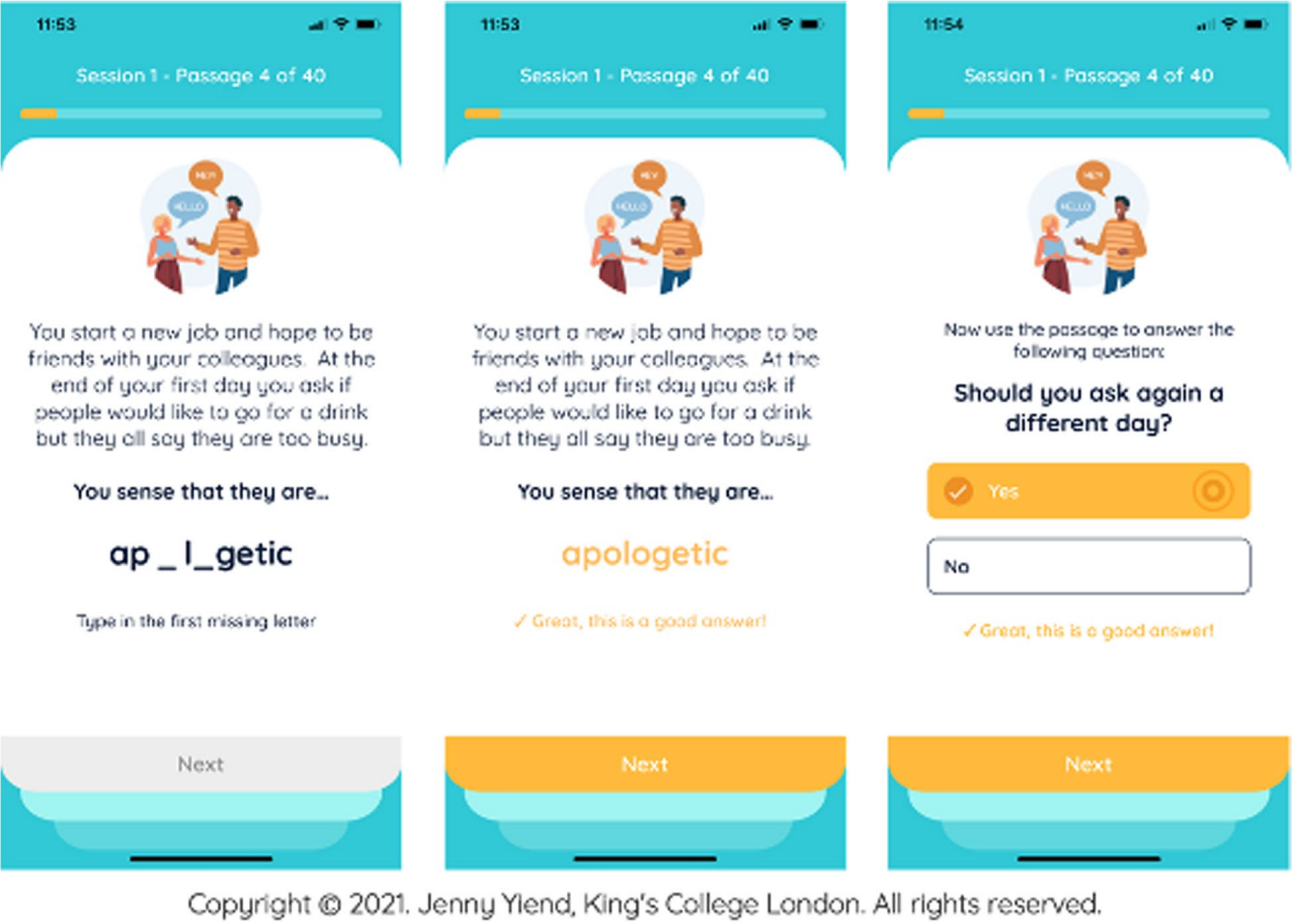
Example of STOP app design and content

**Fig. 2:**
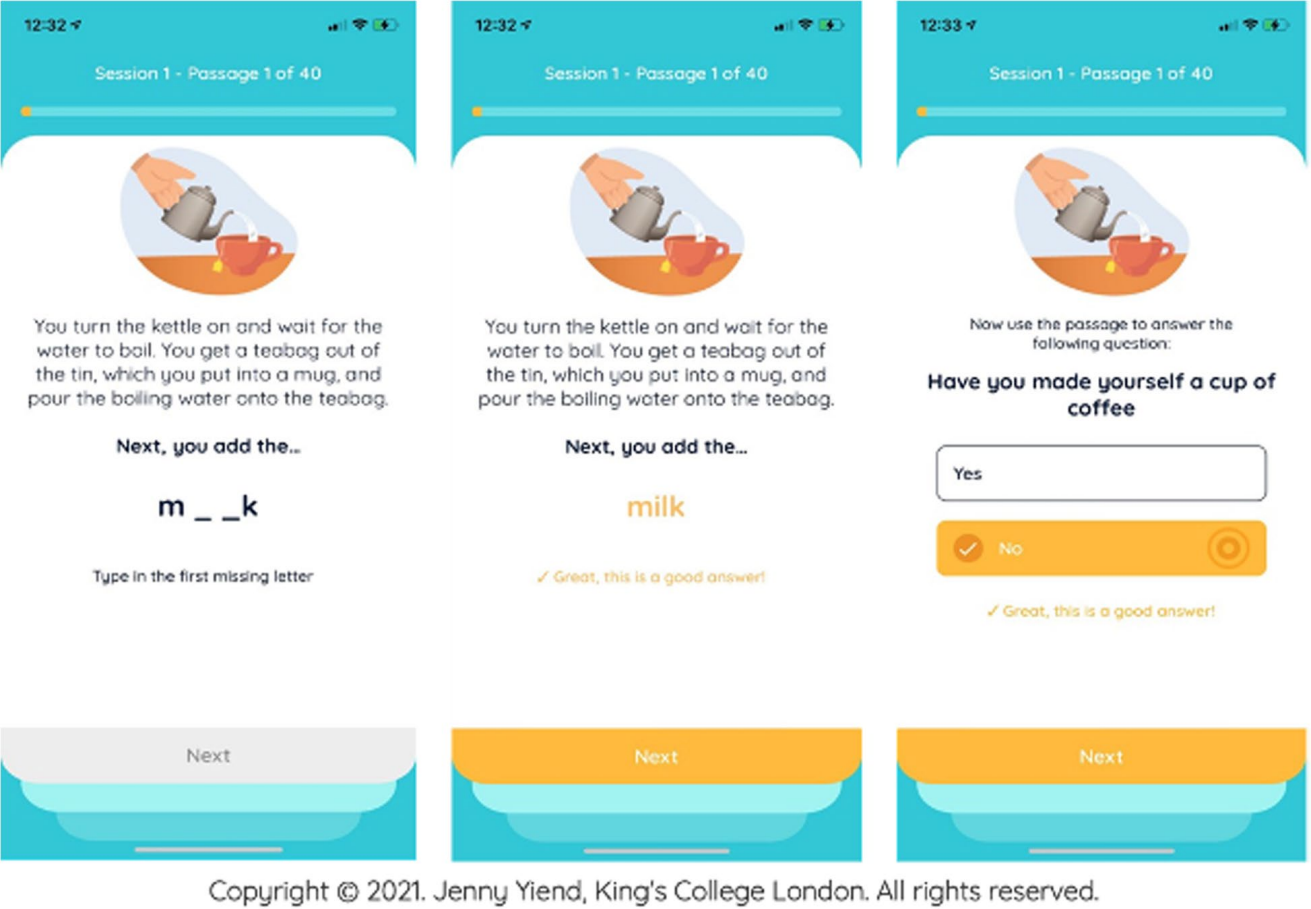
Example of the control app design and content

The STOP app uses a proposed digitally based therapy for paranoia called Cognitive Bias Modification for paranoia (CBM-pa) [26, 27]. Participants read stories in the app and completed missing words and answered questions about each story in a way that encourages more helpful beliefs about themselves and others (Fig. 1). STOP encourages people to develop non-threatening alternative ways of interpreting ambiguous social scenarios, leading participants to reduce their bias towards interpreting such scenarios in a personally threatening way. The control arm of the trial involved reading the same type of stories and performing the same tasks (filling in missing words and answering questions), but without the interventional content designed to change paranoid thinking (Fig. 2). The control app was identical to the STOP app in appearance, features, and functionality; however, the item content that participants saw omits the ‘active ingredient’, control participants read and responded to factual material or mundane everyday experiences. The persuasive features included in the STOP app (and the identical control app) are detailed and described in Table 1.

**Table 1:** The persuasive systems design model with features present in the STOP app explained

Category	Persuasive Feature	Present in STOP/control?	Implementation in STOP app
Primary Task Support		Yes	
	Reduction	Yes	Complex content is simplified into brief daily sessions
	Tunnelling	Yes	Sequential progression through scenario-based learning– scenarios become more paranoia provoking in later weeks
	Tailoring	No	-
	Personalization	No	-
	Self-monitoring	No	-
	Simulation	No	-
	Rehearsal	Yes	App allowed users to practice applying new thinking patterns to real life scenarios
Dialogue Support		Yes	
	Praise	Yes	Positive feedback from to app to user after completing tasks
	Rewards	Yes	Achievement badges awarded for progress
	Reminders	Yes	Push notifications to encourage app use
	Suggestion	No	-
	Similarity	Yes	Scenarios in app designed to remind users of their own lives and experiences
	Liking	Yes	Visual design with appealing interface, colors, images and moving visuals
	Social role	No	-
System Credibility Support		Yes	
	Trustworthiness	Yes	Information in the app is presented in clear, transparent manner
	Expertise	No	-
	Surface credibility	Yes	App has a professional appearance and functionality
	Real-world feel	Yes	App includes information about the developers and research team including their contact details
	Authority	No	-
	Third-party endorsements	No	-
	Verifiability	No	-
Social Support		No	
	Social learning	No	-
	Social comparison	No	-
	Normative influence	No	-
	Social facilitation	No	-
	Cooperation	No	-
	Competition	No	-
	Recognition	No	-

### Participants

For the current study examining digital therapeutic alliance, participants in the STOP trial who attended their 24-week follow-up were informed about further research opportunities. If they agreed they were interested to hear more, their contact details were passed on with consent. Out of 18 participants approached, 13 agreed to participate in this interview study. We recruited participants regardless of which arm of the STOP trial they had been assigned to, as all participants had experienced using the app, albeit with different content depending on their randomization. Given evidence that belief in treatment can lead to alliance formation regardless of whether patients receive an intervention or placebo [28], and that the impact of mental health smartphone apps may extend beyond interventional content to include users’ relationships with their devices [29], this study included participants in both the control and intervention arms. Due to the blinding protocol of the parent trial, we did not have information on how much each participant had used the app or which arm they had been randomized to.

Interested participants received an information sheet, had the opportunity to ask any questions, and gave written informed consent. Purposive sampling of those interested was used in priority order of gender, ethnicity, and age. The researcher conducting the sampling had access only to these demographic characteristics and not to any other data about their participation in the STOP trial, such as level of engagement with the app or which arm, they had been assigned to. This purposive sampling approach was used to interview as diverse a participant group as possible to increase the representativeness and usefulness of the data [30].

A sample size of 12 was proposed based on study characteristics such as sample homogeneity and focused study aims and scope [31, 32]. During data collection, saturation was reached at 13 interviews when additional information obtained from subsequent interviews continued to fit into existing categories [33].

### Patient & public involvement

The study focus was developed collaboratively with a person with lived experience of using digital interventions for their mental health. They then reviewed the study design and research questions and deemed these relevant from a lived experience perspective. The same person subsequently helped develop all study materials to ensure they were easy to understand for participants. During data analysis, two people with lived experience of psychosis were given training on the framework method. They then contributed to the coding of data and construction of themes from these codes, as well as participating in research analysis meetings.

### Data collection

One-off, in-depth, semi-structured interviews were conducted in line with the study protocol developed before the study commenced. Interviews were conducted using Microsoft Teams between September and December 2023. A topic guide (Supplementary Information 1) of questions based on Bordin’s pan-theoretical model of therapeutic alliance [9], persuasive systems design [21], and previous literature on digital therapeutic alliance was used. The topic guide specifically focused on participants’ experiences of connecting with the app, rather than general satisfaction with the app, and included questions designed to explore the quality of the relationship they formed with the app. This was piloted on the first 3 interviews and these interviews were coded to ascertain if the questions were producing relevant data. These pilot interviews were included in the final analysis as no substantial changes to the topic guide were needed. All participants were given a gift card of £15 following the interview. Interviews were recorded and auto-transcribed by Microsoft Teams with transcripts checked and corrected manually by TT who had also conducted the interviews. Field notes were written during or after interviews to record any additional observations.

### Reflexivity

The researchers took a critical realist epistemological position when using framework analysis [34]. This position assumes that reality cannot be fully represented by data; however, interpretation of data by researchers can create structure and meaning in the data [35]. The interpretation is also affected by the researchers’ own knowledge. The research team consisted of TT, a doctorate in clinical psychology trainee; PJ (academic clinical psychologist); SD (computing and information technology researcher); MJTD (person with lived experience); and JY (academic psychologist). TT has experience delivering psychological interventions to people with psychosis. PJ and JY have extensive experience researching psychosis. SD has extensive research experience in digital mental health and digital therapeutic alliance. MJTD has lived experience and has experience in psychosis research design. The authors’ own perspectives, for example that connections can be formed between technology and humans, and knowledge of clinical practice and digital therapeutic alliance, will have influenced choices made throughout the research process. Involvement of people with lived experience in protocol development, topic guide and material development, and coding and data interpretation was a core part of the research in terms of grounding the data and interpretation of findings from multiple perspectives.

### Analysis

Qualitative analysis software NVivo 12 was used to assist with the data analysis process. Framework Analysis [36] was used as it enabled the use of a framework of deductive codes based on Bordin’s pan-theoretical theory of therapeutic alliance and the persuasive systems design model to identify, analyse, and interpret patterns and meaning within qualitative data alongside inductive codes.

Framework analysis involved a five-stage process: (1) familiarisation with the data, which was done by transcribing and listening to recordings; (2) coding of a subset of the data using a priori codes based on Bordin’s [9] pan-theoretical theory of alliance and persuasive system design framework [23] and inductive codes; (3) developing an analytical framework of codes that could be applied to the remaining dataset; (4) indexing stage in which data were inputted into the analytical framework; and (5) charting stage in which summaries were produced of the data within the analytical framework, and mapping and interpreting of the data where themes were created and reviewed until definitive concepts could be produced from the data. This was done as an iterative process moving between stages as needed. Two people with lived experience (including MJTD) blind coded a sample of the data and assisted in indexing and mapping data into themes in a series of meetings with TT. Codes and themes were also reviewed and developed by the wider research team, including all co-authors.

## Results

Thirteen people participated in the study, of which 62% identified as female and 62% labelled their ethnicity as white (Table 2). Most participants (60%) were over age 40 and 92% said they were currently or previously attended mental health services. Just over half of participants (54%) reported having previously received human-delivered therapy. Interviews ranged between 45 min and 1 h in length.

**Table 2:** Demographic information of participants

Variable	% (n)
Age category	
18–29	15% (2)
30–39	15% (2)
40–49	23% (3)
50–59	39% (5)
> 60	8% (1)
Gender	
Male	38% (5)
Female	62% (8)
Transgender	0% (0)
Ethnicity	
Asian or Asian British	0% (0)
Black, Black British, Caribbean or African	15% (2)
Mixed or Multiple Ethnic Groups	15% (2)
White	62% (8)
Other Ethnic Group	8% (1)
Employment status	
Full-time employment	32% (4)
Part-time employment	38% (5)
Unable to work	15% (2)
Unemployed	15% (2)
Marital status	
Cohabiting	15% (2)
Divorced	15% (2)
Married	23% (3)
Other	8% (1)
Single	39% (5)
Currently or previously used Mental Health Services	92% (12)
Previously received human-delivered therapy	54% (7)

### Persuasive systems design features in the STOP and control app

Table 1 describes Oinas-Kukkonen & Harjumaa’s [21] persuasive systems design model, highlighting the features available in the STOP and control app and providing a brief description of how they were implemented. This information provides context about the app’s design that may influence users’ experience of digital therapeutic alliance. Note that control arm participants experienced the same app interface but without the therapeutic content targeting paranoid thinking patterns.

**Fig. 3:**
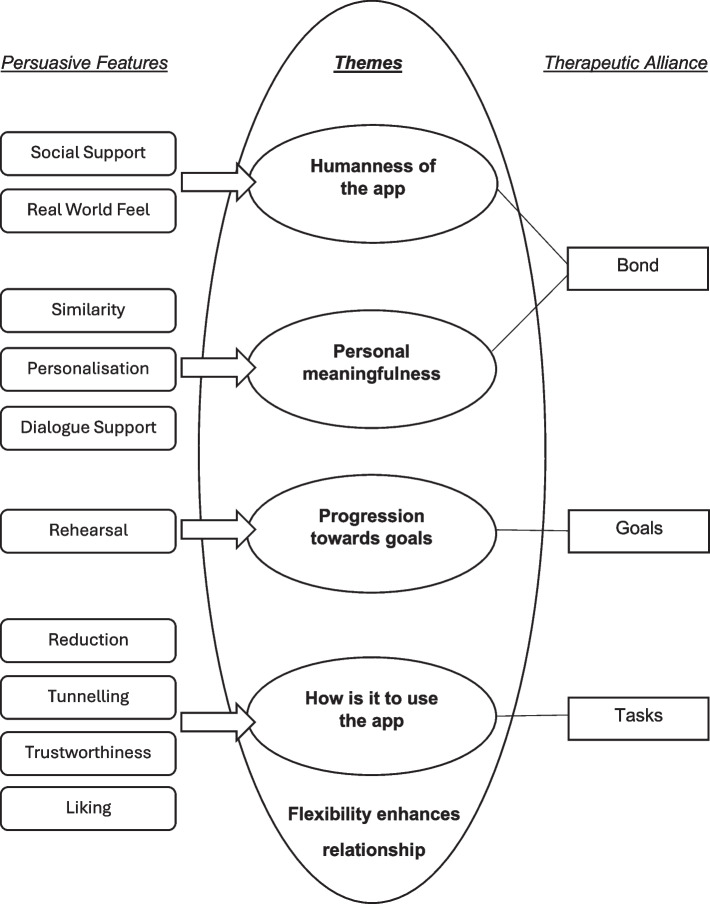
Thematic map of digital therapeutic alliance and its relationship to persuasive features and therapeutic alliance. Note: This figure illustrates how the five themes identified in our analysis (1–5) map onto Bordin’s therapeutic alliance dimensions (Bond, Goals, Tasks), with themes 1–4 being digital analogues of the existing dimensions, and theme 5 providing the unique context for digital therapeutic alliance (as shown in the outer circle). Persuasive features reinforce aspects of digital therapeutic alliance. Not all persuasive features present in the STOP app (as shown in Table 1) were identified by participants as influencing their experience of alliance

Five themes were constructed during data analysis: 1) Humanness of the app, 2) Personal meaningfulness, 3) Progression towards goals, 4) How is it to use the app, and 5) Flexibility enhances relationship. All five themes contribute to the formation of digital therapeutic alliance. Figure 3 shows how these themes map onto therapeutic alliance [9], with themes 1–4 being digital analogues of the existing dimensions of therapeutic alliance and theme 5 (Flexibility enhances relationship) being unique to the digital environment and providing the context for formation of these other themes. Figure 3 also illustrates how persuasive systems design features [21] reinforce and enhance aspects of digital therapeutic alliance.

### Theme 1: Humanness of the app

Although the app is not human, this does not mean it cannot symbolize humans or humanness. Many participants anthropomorphized the app calling it *“friendly” (Participant 3),* saying it had a *“sense of humour”* and *“personality” (Participant 6),* was *“someone to talk to” (Participant 9)* and had “*made me smile” (Participant 2)*. Participants described experiencing a sense of loss when they stopped using the app and expressed wanting a therapeutic ending such as a letter or goodbye conversation.

“I did feel connected with the app because when I finished the trial and then you can’t access [the app] anymore it was a bit like ohh. I felt a bit of a loss.” (Participant 12)

However, for some participants the app’s limitations meant they could not connect with it. Therapeutic abilities of humans including interactiveness, responsiveness, warmth and reassurance were judged beyond the app’s capabilities.

“An app it’s inanimate it’s an object and (my former therapist) was a person a, a person, a person who can challenge the status quo. The app cannot do that.” (Participant 8)

Other participants valued that the app didn’t require the relational intensity human-to-human therapy may involve. They appreciated that the app reduced relational burden, fear of judgment, and treatment didn’t require collecting personal details.

“[The app] feels targeted and it is intensive against [paranoid thoughts] and I think that’s what I really needed… when I’ve been to face to face, they just want to bring up the childhood and trigger stuff and what happened between your parents and blah blah blah” (Participant 9)

Participants reported that the app’s lack of human responsiveness provided more space for personal reflection.

“With a person they’re just speaking, I’d be disagreeing, and with the app, I’ve got more time [to think] well, maybe, wait a minute, maybe this is a bit of paranoia” (Participant 5)

Humanness of the app could also be increased through the app displaying information on app developers or other app users. Most participants reported that Real-World Feel, a persuasive systems design term for the positive impact of a digital system highlighting the people (research assistants/app developers) or organization (the National Health Service) behind the product, built confidence in and connection to the app. This also happened directly through app content. For example, the app contained fun facts which participants said *“[showed] humanity… [which] made me more trusting” (Participant 2)* and *“made me feel a bit more warmer towards it” (Participant 6).*

Furthermore, most participants reported hypothetically that if they had contact with other app users, this would decrease their sense of isolation and impact their alliance with the app through increasing their motivation. In persuasive systems design, features that allow contact between app users fall under the category of Social Support and can take multiple forms including message boards and anonymized user stories.

“I think [seeing other users on the app] would for me impact positively on: ‘These other people think this is useful as well and these other people are going through the same experience of using this app as me’, so to me that would help my motivation and help my connection with the app.” (Participant 12)

Users reported the app reduced isolation by creating a sense of communion with others experiencing paranoia, with participants feeling the app’s existence implied others shared their struggles despite having no actual contact with other users.

“That element of like community in a weird way… I know everyone has, maybe everyone has those kind of thoughts, but it’s just nice to to know like ohh okay this is actually an issue enough for it to be provided inside an app” (Participant 13).

However, some participants felt that connecting with other app users could backfire, leading to mistrust, judgment and ultimately disengagement.

### Theme 2: Personal meaningfulness

To feel connected to the app participants wanted personally meaningful app content and meaningful responsiveness to their individual inputs. Participants who saw their own circumstances in the app’s scenarios experienced these exercises as more useful. This is the persuasive systems design principle of Similarity (the system reminds me of myself) and when participants encountered this, they reported they reflected more on their struggles. Conversely, participants whose worries and lives were not represented reported disconnection and increased isolation.

“The scenarios… were all things about going to work and things that are not relevant to my [life]. I haven’t been to work for 20 years and going to a party, I’ve been to about one party in my life” (Participant 2)

Participants wanted personally meaningful reciprocity from the app, including using the person’s name, adapting app content based on user inputs, or giving users feedback on their progress. This would indicate a genuineness on the part of the app, but also a respect and collaboration as feedback would allow the user to be an active participant in their treatment.

“It’s quite trusting to know that the product that you’re inputting your data into is also sharing that with you, so there’s like an element of transparency” (Participant 13)

In the persuasive systems design framework this links into principles of Personalisation (the app treats user input responsively) and Dialogue Support (the app is interactive through system feedback). However, arbitrary attempts at reciprocity risk rupturing the relationship and promoting disengagement. For example, giving users badges as they progressed (known as Rewards in the persuasive systems framework) which participants said were not reinforcing as they lacked meaning and were not married to personal goals.

“It’s like a gold star at school where you get a gold star at school, but then it’s like, what does the gold star really mean?” (Participant 6)

### Theme 3: Progression towards goals

When the app’s purpose aligned with the user’s hopes or goals for using the technology, and the user experienced progress towards that goal, alliance increased. This occurred even in the absence of there being agreed goals between the app and the user.

“The more you use it, the more you want to use it because it is making a difference… you start thinking a bit more clearly and putting things into perspective” (Participant 3)

Participants who made real-world progress towards goals through practicing first in the app before practicing in their lives (the Rehearsal feature of persuasive systems design) reported increased motivation to keep using the app and connection to it.

“It did definitely change the way I was thinking… I don’t get panicked on the train anymore when I used to have really bad panic attacks or I couldn’t breathe” (Participant 9)

### Theme 4: How is it to use the app

Participants expressed that the app’s sessions (tasks) could trigger positive or negative emotions and affect their hopefulness about achieving their goals. Interactive elements such as sound, pictures, videos, badges, and colours were reported to increase a sense of playfulness, while fun features have the potential to increase connection by lightening the mood, increasing motivation, facilitating learning, or provoking a sense of achievement. Participants reported where the app was more enjoyable, goals also felt more achievable.

“Those cartoons and so it was not only oral it was visual. So I could visualize the things that was happening, what was being asked of me. So it became more positive as to my goal of getting well.” (Participant 8)

The app’s tasks were generally experienced as manageable, however, alliance declined when tasks were challenging or time intensive. This aligns with the persuasive systems design principle of Reduction, that systems should be simplified.

“The voices start disturbing me [when I didn’t understand the questions]… then I get frustrated I just wanna finish it quick” (Participant 11)

A few participants valued repetitiveness as it reduced task demand, however, most reported repetitiveness of tasks made them perceive the app as boring or tiresome and decreased connection. It symbolized for users a lack of bidirectional relationship as the information they entered into the app never triggered a change in treatment trajectory. This aligns to the persuasive principle of Tunnelling in which the system guides users through a process with opportunities for behavioral or attitude change enroute.

Tasks should also be transparent in their purpose as multiple users expressed wondering if the app might be trying to “trick” them.

“I never trusted the technical glitch I always thought this is deliberate. (Participant 2)

Persuasive systems design dictates that systems should provide information that can be perceived by users to be truthful, fair and unbiased (Trustworthiness). Participants commented that responsive technical support could increase perceived credibility.

### Theme 5: Flexibility enhances relationship

Participants expressed that attempting to access public mental health care can lead to feelings of frustration and rejection. However, participants reported that they had a positive relationship with the app as its flexibility and availability allowed them treatment they previously felt barred from.

“I’ve got to really fight for [face to face therapy] whereas the app… it’s my choice” Participant 12)

Participants reported this boosted a sense of autonomy and promoted a bond with the app based on it being there when others were not. Similarly, being able to connect to the app in times of increased paranoia and use the app as a break from aversive experiences increased bond.

“During the time I was using the app I did become quite ill and I had to go into a crisis house and so it did help me distract myself at times when there weren’t workers or anything available” (Participant 5)

For some participants flexibility of the app was meaningless as the app could not provide the type of support they viewed as helpful.

“I get days I’ll get low and and how can the app help me? Whereas I can ring [care coordinator] and tell [care coordinator]: ‘Help can you take me out to do something?’ The app can’t do that and especially for someone like me, I’m not confident person” (Participant 10)

Participants noted that app flexibility could lead to disengagement, while scheduled regular sessions promoted momentum. The app uses strategies such as reminders to keep users working towards goals; however, no participant reported that techniques like reminders could prevent drop out.

“You can get notifications of the emails and stuff, but life gets in the way” (Participant 7)

All participants who were highly engaged with the app reported this was due to internal motivation.

## Discussion

### Summary of findings

This study set out to investigate how users of a self-guided smartphone application conceptualize the digital therapeutic alliance and how their experience of digital therapeutic alliance was supported by persuasive systems features. Persuasive features were examined as a possible mechanism through which digital therapeutic alliance is created given the unique context of the digital environment.

Five themes were constructed during data analysis: 1) Humanness of the app, 2) Personal meaningfulness, 3) Progression towards goals, 4) How is it to use the app, and 5) Flexibility enhances relationship. All five themes contribute to formation of digital therapeutic alliance. Themes 1–4 were understood to map onto existing dimensions of therapeutic alliance while Theme 5 was interpreted as being part of the context of smartphone interventions and supportive to the formation of the other digital therapeutic alliance dimensions. Persuasive features were found to enhance Themes 1–4.

It is important to note that our methodological approach directly influenced these findings. By using framework analysis with deductive coding based on Bordin’s therapeutic alliance theory and persuasive systems design, our analytical lens was primed to identify these specific dimensions. The interview guide, which was structured around these concepts, also shaped participants’ responses. While we incorporated inductive coding to allow new themes to emerge, the pre-existing frameworks inevitably guided our interpretation of the data.

### Contextualizing findings within previous research

Previous research has drawn mixed conclusions about a user’s ability to bond with a self-guided mental health app. Tong et al. [13] argued that there could not be a bond dimension in digital therapeutic alliance due to bond requiring bidirectional connections and being influenced by the life experiences of those involved. The authors of that paper preferred to use the term “connection” to explain interaction between a user and app. One of the complexities of the term “bond” is that it is conceptualized as a two-way process of connection. However, even in a human-delivered therapy dyad, the therapist is using professional techniques and behaviours to facilitate this connection, and maintains boundaries regarding their life and internal world, thus skewing the two-way aspect of the bond. Furthermore, in human-delivered therapy different therapeutic modalities will necessitate different intensities of bonds, indicating that bond can occur along a spectrum. With this in mind, we propose that the digital therapeutic alliance dimensions of Humanness of the App and Personal Meaningfulness map onto the Bond dimension of therapeutic alliance.

Our findings indicate that users of self-guided mental health apps may draw on their perception of the app developers and other users to lend life story/characteristics to the app where it has none, thus facilitating a bond. Persuasive systems design features of Real-World Feel and Social Support appear to enhance this process, indicating that app developers wanting to nourish alliance should give users information on the team and organizations behind the app and create opportunities for app users to experience peer-to-peer community, either directly or indirectly. This is supported by evidence from a systematic review on peer-to-peer interactions in digital interventions for psychosis, which found that those including moderated peer-to-peer contact were more engaging and acceptable to users and appeared to have increased compliance [37]. This study also indicates that the benefits of feeling less isolated and perceiving oneself as part of a community can be built by an app through its own content, without any peer-to-peer contact. This mirrors findings from a trial of the blended app for paranoia which found users viewed animated vignettes as peers, and thus felt more connected, understood and less isolated [38]. This study’s novel contribution is that both direct and implied social connectivity appears to confer benefits to the app-user relationship, promoting alliance.

Where users experienced a positive emotional response to the app, they anthropomorphized it which increased alliance. This echoes studies which showed users assigned human traits to self-guided mental health chatbots [10, 39]. Reactions from users, such as missing the app on therapy termination, confirms the results of a previous qualitative study on an app for psychosis [11], and gives further evidence to the assertion that a user’s reaction to a mental health app is not an emotionally cold response to a piece of technology but can include warmth and be relationally based. This is further validated by findings on smartphone attachment, which indicate that when individuals experience fatigue or stress, they may experience emotional bonding with a mental health app that meets their emotional needs [40]. However, it is not clear if the experience of bond in the digital context would be as strong as with a human therapist as this goes beyond the data available in this study.

This study also found the app’s non-human manner of interacting was experienced by some users as reducing relational burden and fear of judgment. This aligns with previous findings that people could be open and honest with self-guided mental health apps [11] with reduced pressure [13], and that users may be turned off by digital technologies that are experienced as trying too hard to be human [41]. Many users appreciated the app’s focused approach, which may be particularly relevant in a population with significant contact with mental health services. Ambivalence about the utility of connecting with others is likely more elevated in people with paranoia due to this client group experiencing impaired social interactions and altered trust [42]. It is also worth noting that about half of the sample had previously received human-delivered therapy, and this may be relevant in considering their level of satisfaction with an app focused on a single therapeutic target (paranoia), as these users had already experienced a potentially more wide-ranging, interactive and containing therapeutic intervention. Multi-featured apps or apps that attempted to more closely replicate the varied experience of interacting with a human may have produced a very different response from participants. Thus, this supports the contention that mental health technologies provide more than an emulation of human-delivered interactions but provide unique relational and therapeutic opportunities.

This study supports the notion that lack of interactivity and personal responsiveness to individuals impacts alliance [11, 13, 22]. Although an app may not be able to make an empathic body gesture or facial expression, several persuasive features may increase alliance including Personalization (the app adapting to user input such as using their name) and Dialogue Support (interactive elements, feedback or graphs) by giving the user the sensation of a bi-directional collaborative relationship. This study indicated that this feedback and responsiveness can be seen as a sign of respect and genuineness by users and therefore part of the essential relational traits of a therapeutic alliance. However, the digital therapeutic alliance dimension of Personal Meaningfulness in this study contains a warning to those developing apps, that interactive features that lack meaning to users risk promoting disconnection.

Another unique contribution of this study is the idea that where the user perceives the system as being more like themselves (Similarity in persuasive systems design) this will enhance connection. In human-delivered therapy there is substantial evidence that client-therapist match on demographics, life experiences and worldviews influence therapeutic alliance and therapy outcomes [43, 44]. This study indicates that issues of identity and similarity remain relevant online and app developers may want to understand the life experiences of their user group or have different versions of the app for different user groups to build connection (known as Tailoring in persuasive system design).

Previous research suggested that agreed upon goals enhance digital therapeutic alliance in self-guided mental health apps [12, 16, 45]. Our study found that while the app did not allow the user to input a specific, measurable and time limited goal, users who both had a goal in mind which the app aligned with; and saw progress towards this goal, reported increased alliance with the app. This validates Tong et al.’s [13] suggestion that when building alliance with self-guided mental health apps goals may be considered as reasons/goals for using an app as opposed to concrete agreed app-client goals. However, persuasive theory on cognitive consistency and goal setting theory [46] state that setting specific goals increases motivation to continue towards those goals, thus indicating that app developers may benefit alliance by having user stated goals in their interventions. App developers should also note that systems that allow Rehearsal (persuasive systems design principle where things practiced in the app are done in real life) are primed to create more app-user connection as users take progress made in the digital environment and apply this to their lives.

Flexibility is not included in existing therapeutic alliance or digital therapeutic alliance measures; however, it has been proposed in previous literature as being part of the context which allows digital therapeutic alliance to develop [13, 16, 20, 47]. This study found that flexibility has both a surface level (time, location, duration of intervention) and relational impact. Therapeutic contact goes beyond what happens in the therapy session to include a myriad of aspects such as the referral process and waiting times and this study found that the flexibility of an app meant service users did not need to navigate this process and ease of access could mean a more positive relational experience and increased user-app bond. Increased flexibility could also impact the user’s relationship to self, increasing autonomy and empowerment as the user saw themselves as being in the driver’s seat of their treatment. This finding reinforces previous accounts, that the autonomy and self-determination aspects of using digital interventions for mental health treatment may be particularly relevant for those with experiences of serious mental illness [20] often associated with experiencing high chronicity, disability and powerlessness [48, 49]. However, flexibility increases the need for self-initiation of app use and self-motivation as the app lacks robust mechanisms of accountability. This study indicates that app features such as reminders encourage, but do not enforce, app use [13].

This study verified previous assertions that tasks are predictive of digital therapeutic alliance [16, 18, 19] and indicates that an app that is effortful to use can lead to alliance ruptures which the app will not be able to repair due to its reduced communications abilities. Thus, the persuasive systems design principle of Reduction (simplifying complex tasks) is essential in building apps that maintain connection. In therapeutic alliance genuineness is one of the core components in building alliance in face-to-face therapy. Rogers [50] (p. 185) wrote that “genuineness in therapy means that the therapist is his actual self during the encounter with his client. Without facade.” This concept of genuineness, transferred to the context of self-guided mental health apps, could represent whether an app does what it claims to. This study found that users needed to feel sure of what the app was doing and its intentions. The persuasive systems design principle of Trustworthiness is relevant here and app development teams must take steps to boost app credibility in the eyes of the users, for example a simple strategy such as warning users that technical glitches may happen and having prompt technical support may demonstrate genuineness to users.

Future research could explore the impact of design on digital therapeutic alliance, for example by comparing different versions of the same clinical content with varying features. It is currently poorly understood to what degree digital therapeutic alliance is linked with outcomes in apps and thus selecting appropriate methods to assess the association between digital therapeutic alliance and clinical outcomes is important, which is likely to require the development of new measures. Assessing digital therapeutic alliance in a variety of types of self-guided mental health apps would also expand understanding of how this concept applies across different therapeutic approaches, user populations, and clinical presentations.

### Strengths and limitations

The participants recruited in qualitative studies interviewing mental health app users are frequently younger digital natives [51, 52]. A strength of this study is that most participants were aged 40 years or above. This expands understanding to a wider range of users of mental health apps, especially given that an older age group are sometimes subject to stereotypical views that they will not be interested in, or be able to use, digital interventions which could lead to unequal access to new innovations in treatment. Another study design strength was the use of a multidisciplinary study team which also included people with lived experience as this allowed a more nuanced data analysis.

All the participants in this study had experience of using the same app, which was designed to help with paranoia. This had the advantage of creating a more homogenous sample which facilitated the analysis process; however, this does mean that the findings may not be representative of users of a different kind of mental health app. As the sample for this paper was taken from a randomized control trial, the study team were blinded to whether those completing the interview were from the two intervention or one control arm. Thus, this sample is a mix of those who received both intervention and control content and it is not possible to extract the influence of this on the data. Furthermore, length of intervention (either 6 or 12 weeks) may have also impacted on experiences of alliance.

Respondent validation, which includes inviting participants to comment on the interview transcript and whether the final themes and concepts created adequately reflect the phenomena being investigated could have been used to increase the credibility of the findings [53], as well as to potentially generate additional data or analysis which may have added further depth to the findings [54].

## Conclusions

This study provides qualitative support for the existence of a digital therapeutic alliance between a user and a mental health app when there is no therapist involvement. Our data suggests that there are relational aspects to people’s use of mental health apps which appear to be digital analogues of alliance as experienced in human-delivered therapeutic interactions. Dimensions of digital therapeutic alliance proposed by previous authors, such as flexibility, are also confirmed in this study as being of importance. Thus, this convergence of new dimensions across multiple investigations is a promising sign of an increasing understanding of digital therapeutic alliance. This study is also the first to link digital therapeutic alliance to persuasive features which outlines tangible information for app developers who can implement these features with the potential to enhance the relational quality of interventions. Findings from this study could be used to inform the design of digital interventions to enhance their capacity to foster digital therapeutic alliance with users, with the supposition that as with the traditional therapeutic alliance, its digital counterpart is also conducive to better outcomes in terms of mental health app efficacy.

## Supplementary Information

Supplementary Material 1.

## Data Availability

The datasets supporting the conclusions of this article are available in the University of Bath Data Archive repository https://doi.org/10.15125/BATH-01412. (Please note this link will only become live once publication has been agreed. If reviewers wish to view the data ahead of publication a restricted link can be created on request).

## Abbreviations

ARM: Agnew Relationship Measure
DWAI: Digital Working Alliance Inventory
HCI: Human Computer Interaction
mARM: Mobile Agnew Relationship Measure
WAI: Working Alliance Inventory

## References

[1] Bond RR, Mulvenna MD, Potts C, O’Neill S, Ennis E, Torous J. Digital transformation of mental health services. NPJ Ment Health Res. 2023;2(1):13. 38609479 10.1038/s44184-023-00033-y PMC10955947

[2] Torous J, Bucci S, Bell IH, Kessing LV, Faurholt-Jepsen M, Whelan P, Carvalho AF, Keshavan M, Linardon J, Firth J. The growing field of digital psychiatry: current evidence and the future of apps, social media, chatbots, and virtual reality. World Psychiatry. 2021;20(3):318–35. 34505369 10.1002/wps.20883 PMC8429349

[3] Linardon J, Torous J, Firth J, Cuijpers P, Messer M, Fuller-Tyszkiewicz M. Current evidence on the efficacy of mental health smartphone apps for symptoms of depression and anxiety. A meta-analysis of 176 randomized controlled trials. World Psychiatry. 2024;23(1):139–49. 38214614 10.1002/wps.21183 PMC10785982

[4] Baumel A, Muench F, Edan S, Kane JM. Objective user engagement with mental health apps: systematic search and panel-based usage analysis. J Med Internet Res. 2019;21(9):e14567. 31573916 10.2196/14567 PMC6785720

[5] Linardon J, Fuller-Tyszkiewicz M. Attrition and adherence in smartphone-delivered interventions for mental health problems: a systematic and meta-analytic review. J Consult Clin Psychol. 2020;88(1):1–13. 31697093 10.1037/ccp0000459

[6] Baumel A, Kane JM. Examining predictors of real-world user engagement with self-guided eHealth interventions: analysis of mobile apps and websites using a novel dataset. J Med Internet Res. 2018;20(12):e11491. 30552077 10.2196/11491 PMC6315225

[7] Flückiger C, Del Re AC, Wampold BE, Horvath AO. The alliance in adult psychotherapy: a meta-analytic synthesis. Psychotherapy. 2018;55(4):316–40. 29792475 10.1037/pst0000172

[8] Horvath AO, Del Re AC, Flückiger C, Symonds D. Alliance in individual psychotherapy. Psychother. 2011;48(1):9–16. 10.1037/a0022186 21401269

[9] Bordin ES. The generalizability of the psychoanalytic concept of the working alliance. Psychol Psychother Theory Res Pract. 1979;16(3):252–60.

[10] Beatty C, Malik T, Meheli S, Sinha C. Evaluating the therapeutic alliance with a free-text CBT conversational agent (Wysa): a mixed-methods study. Front Digit Health. 2022;4:847991. 35480848 10.3389/fdgth.2022.847991 PMC9035685

[11] Berry K, Salter A, Morris R, James S, Bucci S. Assessing therapeutic alliance in the context of mHealth interventions for mental health problems: development of the Mobile Agnew Relationship Measure (mARM) questionnaire. J Med Internet Res. 2018;20(4):e90. 29674307 10.2196/jmir.8252 PMC5934536

[12] Goldberg SB, Baldwin SA, Riordan KM, Torous J, Dahl CJ, Davidson RJ, Hirshberg MJ. Alliance with an unguided smartphone app: validation of the digital working alliance inventory. Assessment. 2022;29(6):1331–45. 34000843 10.1177/10731911211015310 PMC8599525

[13] Tong F, Lederman R, D’Alfonso S, Berry K, Bucci S. Conceptualizing the digital therapeutic alliance in the context of fully automated mental health apps: a thematic analysis. Clin Psychol Psychother. 2023;30(5):998–1012. 37042076 10.1002/cpp.2851

[14] Shrepp M. User Experience questionnaire handbook: all you need to know to apply the UEQ successfully in your projects (version 11). 2023.

[15] Idrees AR, Kraft R, Pryss R, Reichert M, Baumeister H. Literature-based requirements analysis review of persuasive systems design for mental health applications. Procedia Comput Sci. 2021;191:143–50.

[16] Tong F, Lederman R, D’Alfonso S, Berry K, Bucci S. Digital therapeutic alliance with fully automated mental health smartphone apps: a narrative review. Front Psychiatry. 2022;13:819623. 35815030 10.3389/fpsyt.2022.819623 PMC9256980

[17] Darcy A, Daniels J, Salinger D, Wicks P, Robinson A. Evidence of human-level bonds established with a digital conversational agent: cross-sectional, retrospective observational study. JMIR Form Res. 2021;5(5):e27868. 33973854 10.2196/27868 PMC8150389

[18] Scherer S, Alder J, Gaab J, Berger T, Ihde K, Urech C. Patient satisfaction and psychological well-being after internet-based cognitive behavioral stress management (IB-CBSM) for women with preterm labor: a randomized controlled trial. J Psychosom Res. 2016;80:37–43. 26721546 10.1016/j.jpsychores.2015.10.011

[19] Gómez Penedo JM, Coyne AE, Constantino MJ, Krieger T, Hayes AM, Grosse Holtforth M. Theory-specific patient change processes and mechanisms in different cognitive therapies for depression. J Consult Clin Psychol. 2020;88(8):774–85. 32338931 10.1037/ccp0000502

[20] Tremain H, McEnery C, Fletcher K, Murray G. The therapeutic alliance in digital mental health interventions for serious mental illnesses: narrative review. JMIR Ment Health. 2020;7(8):e17204. 32763881 10.2196/17204 PMC7442952

[21] Oinas-Kukkonen H, Harjumaa M. Persuasive systems design: key issues, process model and system features. In: Howlett M, Mukherjee I, editors. Routledge handbook of policy design. 1st ed. New York: Routledge; 2018.

[22] D’Alfonso S, Lederman R, Bucci S, Berry K. The digital therapeutic alliance and human-computer interaction. JMIR Ment Health. 2020;7(12):e21895. 33372897 10.2196/21895 PMC7803473

[23] Tong A, Sainsbury P, Craig J. Consolidated criteria for reporting qualitative research (COREQ): a 32-item checklist for interviews and focus groups. Int J Qual Health Care. 2007;19(6):349–57. 17872937 10.1093/intqhc/mzm042

[24] O’Brien BC, Harris IB, Beckman TJ, Reed DA, Cook DA. Standards for reporting qualitative research: a synthesis of recommendations. Acad Med. 2014;89(9):1245. 24979285 10.1097/ACM.0000000000000388

[25] Yiend J, Taher R, Fialho C, Hampshire C, Hsu C-W, Kabir T, Keppens J, McGuire P, Mouchlianitis E, Peters E, et al. Assessing the efficacy and safety of STOP (successful treatment for paranoia)—an app-based cognitive bias modification therapy for paranoia: a randomised clinical trial protocol. Trials. 2024;25(1):806. 39623444 10.1186/s13063-024-08570-3 PMC11610111

[26] Leung CJ, Fosuaah A, Frerichs J, Heslin M, Kabir T, Lee TMC, McGuire P, Meek C, Mouchlianitis E, Nath AS, et al. A qualitative study of the acceptability of cognitive bias modification for paranoia (CBM-pa) in patients with psychosis. BMC Psychiatry. 2019;19(1):225. 31337373 10.1186/s12888-019-2215-3 PMC6651961

[27] Yiend J, Lam CLM, Schmidt N, Crane B, Heslin M, Kabir T, McGuire P, Meek C, Mouchlianitis E, Peters E, et al. Cognitive bias modification for paranoia (CBM-pa): a randomised controlled feasibility study in patients with distressing paranoid beliefs. Psychol Med. 2023;53(10):4614–26. 35699135 10.1017/S0033291722001520 PMC10388312

[28] Markowitz JC. Supportive evidence: brief supportive psychotherapy as active control and clinical intervention. Am J Psychother. 2022;75(3):122–8. 35232221 10.1176/appi.psychotherapy.2021.20210041

[29] Torous J, Firth J. The digital placebo effect: mobile mental health meets clinical psychiatry. Lancet Psychiatry. 2016;3(2):100–2. 26851322 10.1016/S2215-0366(15)00565-9

[30] Robinson OC. Sampling in interview-based qualitative research: a theoretical and practical guide. Qual Res Psychol. 2014;11(1):25–41.

[31] Hennink M, Kaiser BN. Sample sizes for saturation in qualitative research: a systematic review of empirical tests. Soc Sci Med. 2022;292:114523. 34785096 10.1016/j.socscimed.2021.114523

[32] Vasileiou K, Barnett J, Thorpe S, Young T. Characterising and justifying sample size sufficiency in interview-based studies: systematic analysis of qualitative health research over a 15-year period. BMC Med Res Methodol. 2018;18(1):148. 30463515 10.1186/s12874-018-0594-7 PMC6249736

[33] Braun V, Clarke V, Hayfield N, Terry G. Thematic analysis. In: Liamputtong P, editor. Handbook of research methods in health social sciences. 1st ed. Singapore: Springer Singapore; 2019. p. 843–60.

[34] Fletcher AJ. Applying critical realism in qualitative research: methodology meets method. Int J Soc Res Methodol. 2017;20(2):181–94.

[35] Willig C. Perspectives on the epistemological bases for qualitative research. In: APA handbook of research methods in psychology: foundations, planning, measures, and psychometrics, vol 1. 2nd ed. Washington, DC: American Psychological Association; 2023. p. 5–22.

[36] Ritchie J, Spencer L. Qualitative data analysis for applied policy research. In: Bryman A, Burgess RG, editors. Analyzing qualitative data. edn. New York: Routledge; 1994.

[37] Biagianti B, Quraishi SH, Schlosser DA. Potential benefits of incorporating peer-to-peer interactions into digital interventions for psychotic disorders: a systematic review. Psychiatr Serv. 2018;69(4):377–88. 29241435 10.1176/appi.ps.201700283 PMC5988432

[38] Greenwood KE, Gurnani M, Ward T, Vogel E, Vella C, McGourty A, Robertson S, Sacadura C, Hardy A, Rus-Calafell M, et al. The service user experience of SlowMo therapy: a co-produced thematic analysis of service users’ subjective experience. Psychol Psychother Theory Res Pract. 2022;95(3):680–700. 10.1111/papt.12393 PMC9873386 35445520

[39] Dosovitsky G, Bunge EL. Bonding with bot: user feedback on a chatbot for social isolation. Front Digit Health. 2021;3:735053. 34713203 10.3389/fdgth.2021.735053 PMC8526729

[40] Li J, Zhang C, Li X, Zhang C. Patients’ emotional bonding with MHealth apps: an attachment perspective on patients’ use of MHealth applications. Int J Inf Manage. 2020;51:102054.

[41] Venning A, Herd MCE, Oswald TK, Razmi S, Glover F, Hawke T, Quartermain V, Redpath P. Exploring the acceptability of a digital mental health platform incorporating a virtual coach: the good, the bad, and the opportunities. Health Inform J. 2021;27(1):1460458221994873. 10.1177/1460458221994873 33601947

[42] Gromann PM, Heslenfeld DJ, Fett A-K, Joyce DW, Shergill SS, Krabbendam L. Trust versus paranoia: abnormal response to social reward in psychotic illness. Brain. 2013;136(6):1968–75. 23611807 10.1093/brain/awt076

[43] Wintersteen MB, Mensinger JL, Diamond GS. Do gender and racial differences between patient and therapist affect therapeutic alliance and treatment retention in adolescents? Prof Psychol Res Pract. 2005;36(4):400–8.

[44] Chao PJ, Steffen JJ, Heiby EM. The effects of working alliance and client-clinician ethnic match on recovery status. Community Ment Health J. 2012;48(1):91–7. 21681459 10.1007/s10597-011-9423-8

[45] Prochaska JJ, Vogel EA, Chieng A, Kendra M, Baiocchi M, Pajarito S, Robinson A. A therapeutic relational agent for reducing problematic substance use (Woebot): development and usability study. J Med Internet Res. 2021;23(3):e24850. 33755028 10.2196/24850 PMC8074987

[46] Latham GP, Locke EA, Fassina NE. The high performance cycle: standing the test of time. In: Psychological management of individual performance. edn. 2002. p. 199–228.

[47] Clarke J, Proudfoot J, Whitton A, Birch M-R, Boyd M, Parker G, Manicavasagar V, Hadzi-Pavlovic D, Fogarty A. Therapeutic alliance with a fully automated mobile phone and web-based intervention: secondary analysis of a randomized controlled trial. JMIR Ment Health. 2016;3(1):e10. 26917096 10.2196/mental.4656 PMC4786687

[48] Hoffman L, Wisniewski H, Hays R, Henson P, Vaidyam A, Hendel V, Keshavan M, Torous J. Digital opportunities for outcomes in recovery services (DOORS): a pragmatic hands-on group approach toward increasing digital health and smartphone competencies, autonomy, relatedness, and alliance for those with serious mental illness. J Psychiatr Pract. 2020;26(2):80. 32134881 10.1097/PRA.0000000000000450 PMC7135933

[49] Linhorst DM. A history of powerlessness. In: Linhorst DM, editor. Empowering people with severe mental illness: a practical guide. edn. New York: Oxford Academic; 2005. p. 12–39.

[50] Rogers C. Client-centered therapy. In: Arieti S, editor. American handbook of psychiatry, vol. 3. New York: Basic Books; 1966. p. 183–200.

[51] Aref-Adib G, O’Hanlon P, Fullarton K, Morant N, Sommerlad A, Johnson S, Osborn D. A qualitative study of online mental health information seeking behaviour by those with psychosis. BMC Psychiatry. 2016;16(1):232. 27400874 10.1186/s12888-016-0952-0 PMC4940927

[52] Bucci S, Morris R, Berry K, Berry N, Haddock G, Barrowclough C, Lewis S, Edge D. Early psychosis service user views on digital technology: qualitative analysis. JMIR Ment Health. 2018;5(4):e10091. 30381280 10.2196/10091 PMC6236205

[53] Noble H, Smith J. Issues of validity and reliability in qualitative research. Evid Based Nurs. 2015;18(2):34. 25653237 10.1136/eb-2015-102054

[54] Lindheim T. Participant validation: a strategy to strengthen the trustworthiness of your study and address ethical concerns. In: Espedal G, JelstadLøvaas B, Sirris S, Wæraas A, editors. Researching values: methodological approaches for understanding values work in organisations and leadership. edn. Cham: Springer International Publishing; 2022. p. 225–39.

