# Supplementary material for: How do users of a mental health app conceptualise digital therapeutic alliance? A qualitative study using the framework approach
Source: BMC Public Health. 2025 Jul 14;25:2450. doi: 10.1186/s12889-025-23603-5 (PMC12257784; doi:10.1186/s12889-025-23603-5)
Supplement: Supplementary file 1 — Supplementary Material 1. [file 12889_2025_23603_MOESM1_ESM.docx]

**Supplementary Material 1**

Topic guide for semi structured interview

**Introduction**

Thank you for agreeing to take part in this interview. It should take up to 60 minutes, can I check you are happy to go ahead? As you know I’m hoping that the conversations we have will tell us more about how you experienced working the STOP App. I’m very grateful for your time today. I’ll explain how the interview will work and then you can ask any questions you have before we start. The interview will be recorded over Microsoft Teams, and it will also produce a transcription as we go along, this means a written recording of what we are saying. We keep all interviews secure and only members of the study team will have access to them. Once we have checked the transcripts for accuracy the recordings will be deleted, and transcripts will be anonymised. Everything you say is confidential. The only reason we would have to break confidentiality is if we are worried about your immediate safety. We would always talk to you first. If you would like to receive a copy of the results of the research let me know and I can email you that. We will use short quotes from some interviews, but we will always make sure to remove identifying information. After the study is completed, the anonymised transcripts will be stored in a data archive and may be used for further research. The interview will involve talking about your experience of working with the STOP App and how connected/what working relationship you had with that app. Throughout these questions consider the app’s features, for example its videos, activities, assessments, word tasks, questions**,** content, interface, menus, calendar, reminders, colour scheme, branding/logo, badges, trivia etc. You do not have to answer any questions you are uncomfortable with; you can stop the interview at any time and withdraw, take a break, or continue at another time. You can take your time with the questions; it can take time to reflect and that’s okay. I might sometimes ask if you’d like more time. At times I may interrupt you because there is something I want to know more about or am curious about. I hope that is okay. Do you have any questions?

**Was the STOP app helping you to do things that were working towards your mental health goals?**

- Did the exercises it asked you to do seem suited to your thinking patterns, behaviours and life experiences? How did that impact your connection/working relationship with the app?
- Were the things you were asked to do in the app manageable and meaningful in achieving your goals? How did the app do this? (For example, features, language, breaking things down)
- Did any features of the app encourage you to keep working towards your goals?

**Did you trust the app?**

- Was there anything the app did or said that made you feel this was an app with expertise?
- Did the app look and feel credible?
- How did this impact on your connection/working relationship with the app?

**Was the app able to motivate you to continue when you may have discontinued treatment?**

- How did the app do this (for example praising you, tracking your progress reminding you to do sessions?
- How did this impact your connection/working relationship with it?

**In this app there was not any opportunity in the app to connect with others with similar experiences or users of the app. For example, a forum or chat function where you could speak to other users of the app, read the anonymized stories of others who found the app helpful, or a leader board where you could compare your use of the app with others.**

- Is that something you found helpful for your connection or working relationship with the app?

**Have you had therapy before with a human being?**

**Why did you choose mental health apps instead of seeing therapist?**

- Was there anything the app was able to provide that a human therapist might not have?
- What do you think are the differences between your connection/working relationship with the therapist and this app?
- What do you think are the similarities between your connection/working relationship with the therapist and this app?
- Would you describe what you experienced with the app as a relationship/connection?
- Would you want to see a therapist and use the app simultaneously?
- How would you experience working alone with the app without a person being involved?

**Was there anything else you wanted to discuss today?**

- How did you find this interview?
- Any questions for me?
- I know there’s been some emotional things we have discussed. To help you transition to the next part of the day I want to check how you are doing. I’m going to send you the debrief form with some ideas of resources to look at following our call.
- I will send your voucher and debrief sheet
- Do they want to be informed of the results of the study?
